# Sustaining training effects through physical activity coaching (STEP): a randomized controlled trial

**DOI:** 10.1186/s12966-023-01519-w

**Published:** 2023-10-10

**Authors:** Matthias Loeckx, Fernanda M. Rodrigues, Astrid Blondeel, Stephanie Everaerts, Wim Janssens, Heleen Demeyer, Thierry Troosters

**Affiliations:** 1 Department of Rehabilitation Sciences, KU Leuven, Leuven, Belgium; 2 Respiratory division, University Hospitals Leuven, Leuven, Belgium; 3 Department of Medicine, Western of Sao Paulo University (UNOESTE), Guarujá, Brazil; 4 Department of Chronic Diseases and Metabolism, KU Leuven, Leuven, Belgium; 5 Department of Rehabilitation Sciences, Ghent University, Gent, Belgium

**Keywords:** Physical activity, Chronic obstructive pulmonary disease, Rehabilitation and telemedicine

## Abstract

**Background:**

Pulmonary rehabilitation (PR) programs improve physical fitness, symptoms and quality of life (QoL) of patients with COPD. However, improved physical activity (PA) is not guaranteed after PR and the clinical benefits fade off after PR discharge. We aimed to investigate whether a 9 months PA-telecoaching program is able to improve PA of patients with COPD, after 3 months of PR and if this leads to maintenance of PR-acquired benefits.

**Methods:**

Patients with COPD enrolled in a 6-month PR program were randomized to a (semi-automated) PA-telecoaching program or usual care, 3 months after PR initiation. The intervention consisted of a smartphone application with individual targets and feedback (for 6 months) and self-monitoring with a step counter (for 9 months). Patients were followed up for 9 months after randomization. Primary outcome was PA (daily step count by accelerometery), secondary outcomes were exercise tolerance, quadriceps force, dyspnea and QoL.

**Results:**

Seventy-three patients were included (mean ± SD: 65 ± 7 years, FEV_1_ 49 ± 19%, 6MWD 506 ± 75 m, PA 5225 ± 2646 steps/day). The intervention group presented a significant improvement in steps/day at every visit compared to usual care (between-group differences mean ± SE: 1431 ± 555 steps/day at 9 months after randomization, p = 0.01). Secondary outcomes did not differ between the groups.

**Conclusion:**

The semi-automated PA-telecoaching program implemented after 3 months of PR was effective to improve the amount of PA (steps/day) during PR and after follow-up. However, this was not accompanied by the maintenance of other PR-acquired benefits.

**Trial registration:**

ClinicalTrials.gov. Identifier: NCT02702791. Retrospectively registered on March 9, 2016. Start study October 2015. https://clinicaltrials.gov/ct2/show/NCT02702791?term=NCT02702791&draw=2&rank=1.

**Supplementary Information:**

The online version contains supplementary material available at 10.1186/s12966-023-01519-w.

## Background

Multidisciplinary pulmonary rehabilitation (PR) programs result in important and clinically relevant improvements for patients with symptomatic COPD, such as improved functional exercise tolerance, muscle force, symptoms of dyspnea and quality of life (QoL) [1]. Unfortunately, these effects are short-term and tend to wear off in the majority of patients after PR-discharge [2, 3]. The lack of maintenance of these clinical benefits might be linked to a continued lack of physical activity (PA) upon completion of PR. Indeed, patients with COPD, even in early stages of the disease, present decreased PA [4–6] and PA does not restore spontaneously after PR [7]. Therefore, the American Thoracic Society (ATS) and European Respiratory Society (ERS) statement on PR highlights the promotion of PA as one of the goals of PR as long-term health-enhancing behavior [8].

Programs aiming to improve PA outside a PR-setting, using self-monitoring and feedback strategies (i.e. step counters) have been shown effective in several (short-term) trials [9–12]. Recently, such PA promotion programs have been implemented as part of a PR-program, however, with variable success and mostly focusing on short-term PA improvements [13–17]. A reason for lack of success might be the timing to start such programs. As PA interventions are more effective in patients with a better functional capacity, we speculate that such interventions may be more effective when initiated after an initial intensive PR-phase that increases exercise tolerance [7]. One recently published randomized controlled trial (RCT) starting PA coaching at the end of a three-week inpatient-PR program showed an increased PA 6 months later [18].

Furthermore, the effect on the maintenance of PR-acquired benefits of programs using wearable technology to promote PA have not been explored in depth yet. It is tempting to speculate that enhanced PA may indeed be crucial to maintain the benefits of a PR-program.

We conducted a RCT which aimed to investigate (1) Whether implementation of a (semi-automated) PA-telecoaching program, started after 3 months of PR, was effective at increasing PA after 9 months of follow-up (i.e. the long-term effects) and (2) If this PA program resulted in maintained PR-acquired benefits in terms of physical fitness (exercise tolerance, skeletal muscle strength), symptoms and health-related QoL.

## Methods

### Subjects

Patients with COPD who enrolled in the multidisciplinary, six months outpatient PR at the Leuven Hospital were screened for eligibility. After completing three months of PR and after one-week PA assessment, patients were invited to participate to the STEP (‘Sustaining Training Effects through Physical activity promotion’) trial. Main inclusion criteria included being older than 40 years, having cognitive ability to work with electronic devices and absence of comorbidities that interfere with the normal biomechanical movement patterns and therefore PA performance. Further details are outlined in the additional file 1.

### Design

**Fig. 1:**
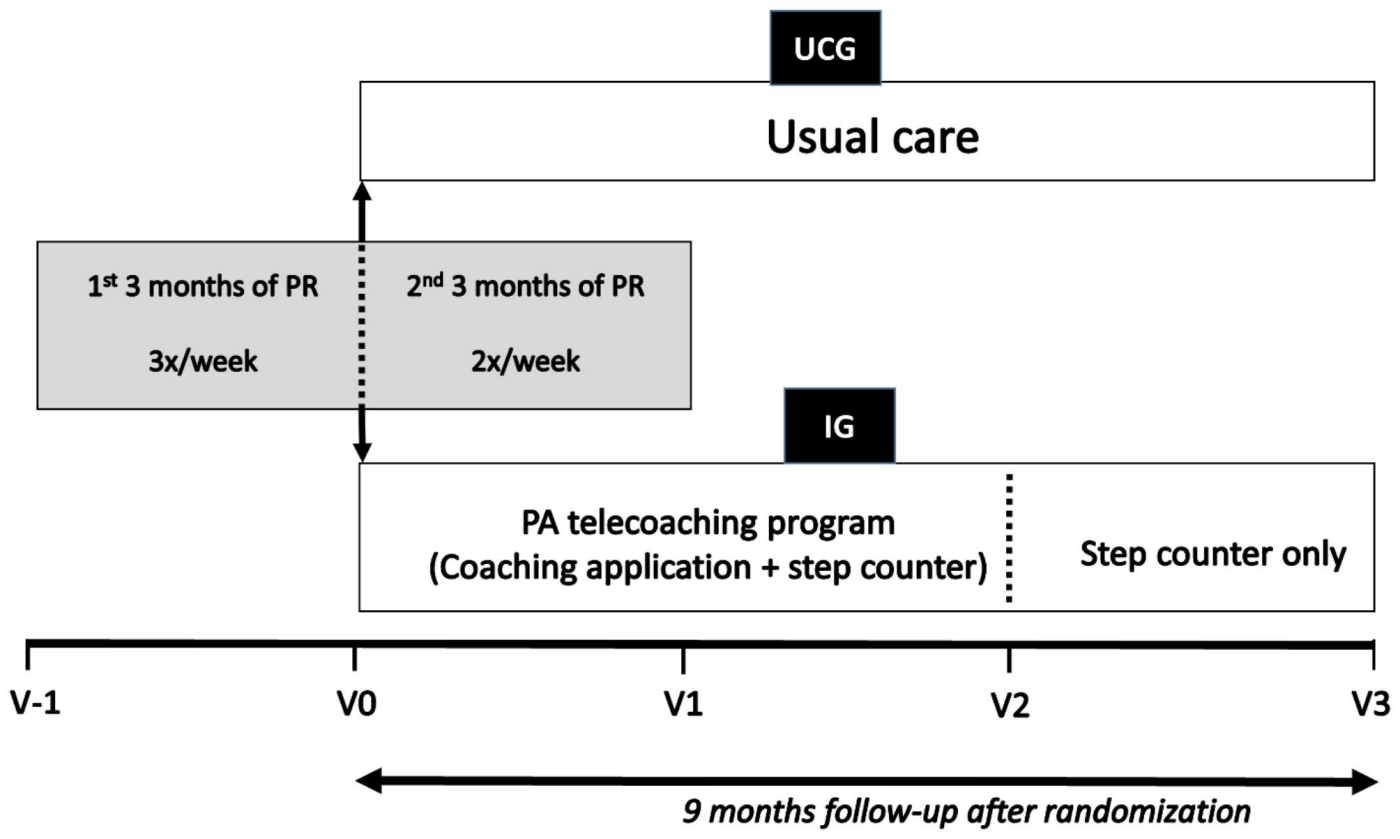
Study design. Four study visits: a visit at randomization (i.e. after 3 months of PR; V0), a visit at the end of PR (i.e. after 6 months of PR; V1), a visit after 3 months of follow-up from PR (V2) and a visit after 6 months of follow-up from PR (V3). Data at the start of PR (i.e. 3 months before randomization) were also used for analysis (V-1). Patients were followed up for 9 months after initiation of the PA telecoaching program. PR, pulmonary rehabilitation; V, visit; UCG, usual care group; IG, intervention group

This was a mono-center, 1:1 prospective RCT (details of randomization outlined in additional file 1). At three months of the 6 months PR-program, eligible patients were included in the study after signing a written informed consent. The trial was registered at clinicaltrials.gov (NCT02702791) and was approved by the local ethics committee (S57963). It consisted of four visits: (1) a visit at randomization, upon completion of 3 months of PR (i.e. start of the study – V0), (2) a visit at the end of PR (i.e. upon completion of 6 months of PR and 3 months after randomization – V1), (3) a visit 3 months after discharge from PR and 6 months after randomization (V2) and (4) a visit 6 months after discharge from PR and 9 months after randomization (V3) (Fig. 1). At the start of PR (i.e. 3 months prior to randomization – V-1), clinical data were assessed as part of PR routine assessments. Randomization took place after completing the first three months of rehabilitation in order to allow the focus on enhancing physical fitness in the first part of the rehabilitation process [19].

The first randomization visit for the first patient took place on October 2015. Last patient last visit took place on September 2019.

### Interventions

#### Pulmonary rehabilitation program (PR)

The standard six-months multidisciplinary program of the Leuven Hospital, previously described in detail [20], was the setting for this study. Patients allocated to the intervention (IG) and usual care groups (UCG) underwent the same PR-program and PR health care providers were blinded to the randomization assignment. Exercise sessions were delivered at a frequency of three times a week in the first three months and twice a week in the second. If patients continued some form of exercise training after PR-discharge, this information was recorded in their file.

### Usual Care

At randomization, patients in the UCG received an information leaflet explaining the importance of and tips on how to become more physically active. Furthermore, at V0, an interview was conducted to assess the level of the patients’ motivation and self-efficacy towards increasing and maintaining their PA level throughout the trial (likert-type scale ranging from 0 (lowest) to 10 (highest)).

### Intervention Group

On top of usual care, patients in the IG received a PA-telecoaching program very similar to the one used previously (NCT02158065–MrPAPP trial), which was proven effective on short-term PA [9, 21]. This intervention consisted of a step counter worn at the waist which provided direct feedback on the number of steps/day patients performed and a project-tailored, semi-automated coaching application on a smartphone (Linkcare® application, Barcelona, Spain) providing daily goal setting, weekly feedback and educational messages. Further details regarding the coaching intervention can be found in the additional file 1.

At randomization, in addition to the assessment of motivation and self-efficacy, the interview in the IG elaborated on barriers of being active, personalized strategies to become more active and a specific action plan was agreed and delivered to the patients, so they could consult it during the intervention.

### Assessments

Prior to each visit, PA was objectively assessed with the Dynaport Movemonitor (DAM, McRoberts BV, The Hague, the Netherlands) and the Actigraph GT3x (ACT, Actigraph LLC Pensacola, Florida, USA), two validated accelerometers(22, 23). As recommended(24), patients were instructed to wear the monitors for seven consecutive days during waking hours. Day-by-day data were exported using the company’s algorithms to retrieve wearing time (DAM and ACT), steps per day (DAM and ACT), movement intensity during walking (MI - DAM) and time in at least moderate intensity activity (MVPA - DAM and ACT). All valid weekdays (at least 8 h of wear time in each) were included in the analyses. A PA assessment was judged adequate and representative if it included at least 2 valid days [24]. DAM was a priori chosen as the primary PA monitor. In case of missing PA data from DAM (e.g. due to technical problems), ACT data were used as input data for multiple imputation to be as complete as possible. Patients did not attend supervised PR-sessions during the PA assessments.

For patient characterization, spirometry, lung volumes and diffusion capacity of the lung for carbon monoxide were measured at V0. Secondary outcomes were (measured at V0, V1, V3) **(1)** maximal exercise tolerance (cycling cardiopulmonary exercise testing (CPET)), **(2)** endurance tolerance (endurance cycling test at 75% of the maximal workload achieved on the CPET at V0), **(3)** functional exercise tolerance (six minutes walking distance(6MWD)), **(4)** quadriceps force (QF) (isometric maximal voluntary contraction against a fixed strain gauge. Results were normalized for body weight (Nm/kg)); and **(5)** symptoms of dyspnea (by modified Medical Research Council(mMRC) for dyspnea) and Qol (Chronic respiratory disease questionnaire(CRDQ)) [25]. 6MWD and QF were additionally assessed at V2.

Exploratory outcomes (measured at V-1 and V3) included **(1)** body composition (total body mass, total body T-score and femoral neck T-score) from a dual energy X-ray absorptiometry (DEXA) scan) and **(2)** Fasting venous blood analysis for levels of glucose, insulin, triglycerides, high-density lipoprotein (HDL) and low-density lipoprotein (LDL). The occurrence and severity of acute exacerbations as well as adverse events were collected during the trial. Further details on all measurements can be found in the additional file 1.

### Statistical analysis

Analyses were performed using SAS Software version 9.4. Values were expressed as mean ± standard deviation (SD) or median [percentile 25-percentile 75] (in case of non-normal distribution – Shapiro-Wilk test) or as numbers (%) in case of frequencies. Statistical significance was set a priori as p < 0.05 for all analyses. All randomized patients were invited for follow-up assessments and included in the Intention-to-treat analysis, even if they stopped using the intervention. The overall effects of the PR-program from V-1 to V0 were descriptively calculated for all patients from assessments obtained in clinical routine.

First, to assess the effect of the PA-telecoaching program on change in PA (i.e. steps/day, MVPA and MI), mixed model analyses were used retrieving the interaction effects at each visit as main results. PA at V0 was chosen as reference. The models were adjusted for daylight as a proxy for seasonality [26]. Multiple imputation through chain Eq. (20 times) was performed in case of missing values of DAM. Day-by-day data of steps and MVPA which were considered to be missing at random were imputed using regression models based on ACT data (proc mi). A weekly average was calculated per multiple imputation dataset and used in further analyses. As a sensitivity analysis, we repeated the main PA analyses based on the complete DAM dataset.

Second, to analyze the effect of the PA-telecoaching program on change of PR-acquired benefits similar mixed models were used for secondary outcomes (maximal, endurance and functional exercise tolerance, quadriceps force, symptoms of dyspnea and QoL). The change in exploratory outcomes (body weight, bone mineral density, blood glucose, lipids and insulin profile) from V-1 (start PR, i.e. 3 months before randomization) to V3 were analysed via ANCOVA analyses, adjusting for values at randomization (V0).

Finally, we explored the difference in proportion of patients with long-term success between UCG and IG using chi-square analyses. Long-term success was defined as an increase above the minimal important difference (MID) at V3 as compared to start of PR (V-1) in terms of (A) functional exercise tolerance (i.e. Δ6MWD ≥ 30 m), (B) symptoms of dyspnea (CRDQ score ≥ 2.5 points) and (C) QoL(CRDQ score ≥ 10 points) [27].

### Sample size calculation

Sample size calculation was based on the primary research question (i.e. effectiveness of the intervention on daily step count). To obtain a power of 80%, expecting a drop-out rate of 20%, a total of 82 patients needed to be included in this study (based on a between group difference of 1785 steps/day, SD of 2505 and α-level of 0.05). These data were based on preliminary results of the tele coaching study running in our center [9]. Because of the slower than expected recruitment, the power analysis was recalculated based on the final results of the tele coaching study [9] and the actual drop out (12%). Therefore, recruitment was stopped at 73 patients (power 80%, alpha 0.05).

## Results

### Patient characteristics

**Fig. 2:**
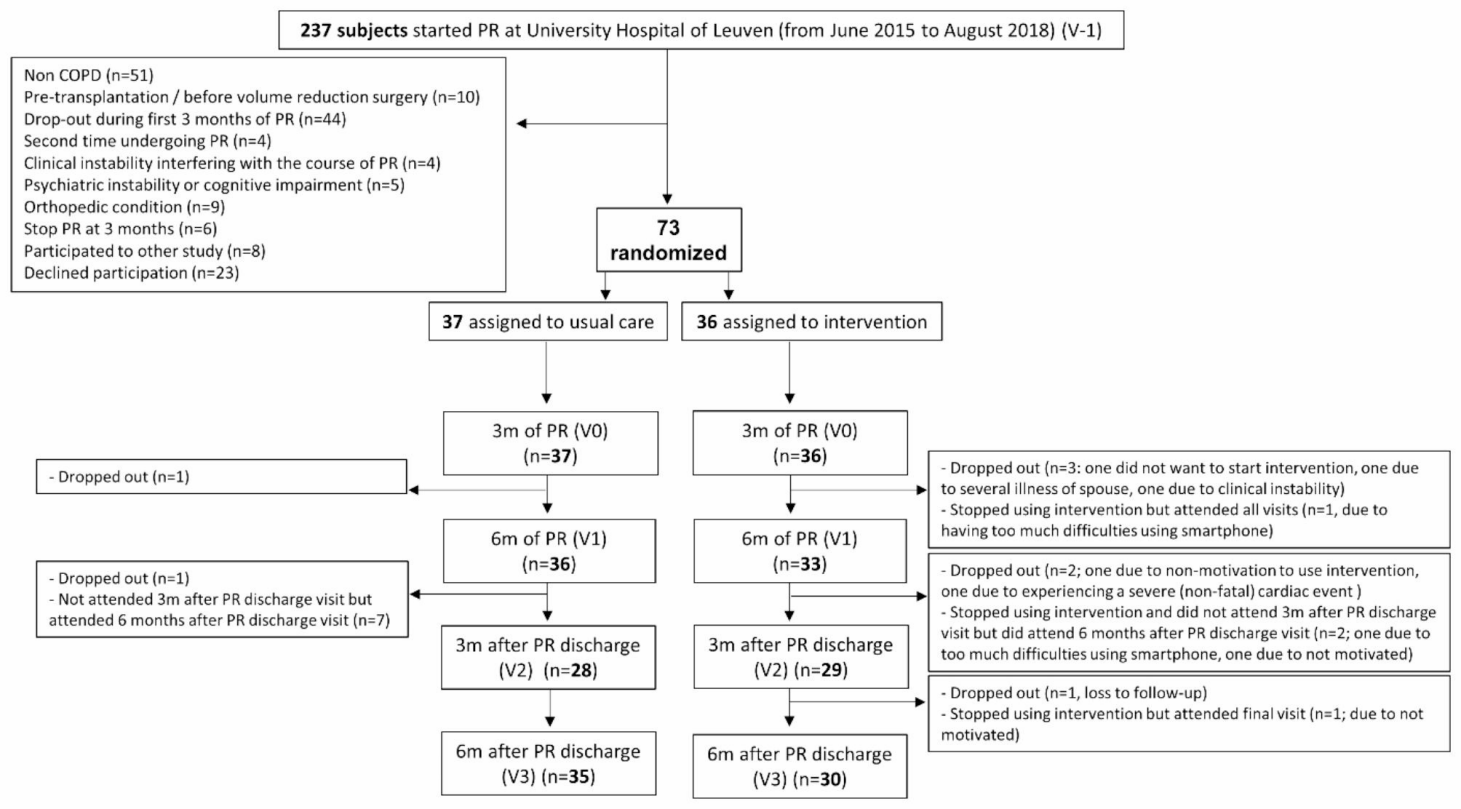
Flow diagram of STEP trial. Numbers refer to the total number of patients evaluated at each timepoint, independently of obtaining valid physical activity measures. PR, pulmonary rehabilitation program; COPD, Chronic Obstructive Pulmonary Disease; m, months; V, visit. Each visit (i.e. V-1, V0, V1, V2, V3) has an interval of 3 months in between

In total, 73 patients were randomized to either the UCG (n = 37) or IG (n = 36) after 3 months of PR, of which 65 were followed up until the end of trial (Fig. 2). Patients’ characteristics at randomization (V0) are displayed in Table 1 and Additional file 2. Patients in both groups had a low PA (mean 5225 ± 2646 steps/day), a modestly reduced exercise tolerance (6MWD 80 ± 12% predicted normal) but QF returned to expected normal values (99 ± 26% of predicted normal value) [28]. The randomized patients wore the DAM for a mean of 4.82 ± 0.46 weekdays with a mean wearing time of 872 ± 98 min/day. Patients included in both groups presented similar high levels of motivation to increase PA (9 [8–10] out of 10 for both groups, p = 0.34) and self-efficacy to become more physically active scores of 8 [6–9] out of 10 in the UCG and 8 [7–9] out of 10 in the IG (p = 0.75).

**Table 1:** Patient characteristics at randomization

	UCG (n = 37)	IG (n = 36)	All (n = 73)
**Age (years)**	66 ± 8	62 ± 7	65 ± 7
**Gender [n (% male)]**	20 (54%)	22 (61%)	42 (58%)
**BMI (kg/m** ^**2**^ **)**	26 ± 5	24 ± 6	25 ± 5
**FEV**_**1**_ **(% pred)**	49 ± 17	50 ± 21	49 ± 19
**TL**,_**CO**_**(% pred)**	50 ± 18	50 ± 14	50 (16)
**VO**_**2**_ **peak (ml/min/kg)**	15.9 ± 4.2	18.4 ± 4.0	16.8 ± 4.2
**VO**_**2**_ **peak (% pred)**	77 ± 23	78 ± 42	77 ± 33
**Endurance time CWRT (s)**	321 ± 143	374 ± 212	345 ± 179
**6MWD (m)**	499 ± 72	512 ± 79	506 ± 75
**6MWD (% pred)**	81 ± 12	79 ± 11	80 ± 12
**Max isom QF (Nm)**	137 ± 41	133 ± 52	135 ± 47
**Max isom QF (Nm/kg)**	1.83 ± 0.47	1.94 ± 0.55	1.89 ± 0.51
**CRDQ**_**dyspnea (points)**_	22 ± 6	20 ± 5	21 ± 6
**CRDQ**_**total (points)**_	93 ± 15	89 ± 12	91 ± 14
**PA (steps/day)**	5530 ± 2910	4910 ± 2352	5225 ± 2646
**Time spent in MVPA (min/day)**	87 ± 40	80 ± 31	83 ± 36
**Movement intensity (m/s** ^**2**^ **)**	1.68 ± 0.26	1.78 ± 0.21	1.73 ± 0.24
**Daylight (min/day)**	763 ± 183	760 ± 188	761 ± 184
**Wearing time (min/day)**	873 ± 95	870 ± 102	872 ± 98
**Valid weekdays (n)**	4.85 ± 0.44	4.78 ± 0.49	4.82 ± 0.46

Values presented as mean ± standard deviation or as number (percentage). UCG, usual care group; IG, intervention group; BMI, n, number of patients; body mass index; kg, kilograms; m,meters; FEV_1_, forced expiratory volume in 1 s; pred, predicted; FEV_1_/FVC, tiffeneau index; TL_CO_, diffusing capacity for carbon monoxide; ; VO_2_, oxygen uptake; ml, millilitres; min, minutes; CWRT, constant work rate test; s, seconds; 6MWD, six minutes walking distance; Max, maximal; isom, isometric; QF, quadriceps force; Nm, Newtonmeters; CRDQ, Chronic Respiratory Disease Questionnaire; PA, physical activity; MVPA, moderate to vigorous PA.; PA data is based on DAM data (n = 65)

Characterization of patients at the start of PR (V-1, 3 months before randomization) is included in the Additional file 3. Overall, changes in PA in the first 3 months of PR (V-1 to V0) were modest (Δ725 ± 1822 steps/day and Δ8 ± 24 min/day MVPA). As expected, patients experienced, at the group level, prior to randomization (V-1 to V0) meaningful improvements in maximal and functional exercise tolerance and Qol (ΔWork rate (WR) = 10 ± 115 Watts, Δ6MWD = 43 ± 28 m and ΔCRDQ total = 14 ± 12 points).

### Intervention effect on physical activity

**Fig. 3:**
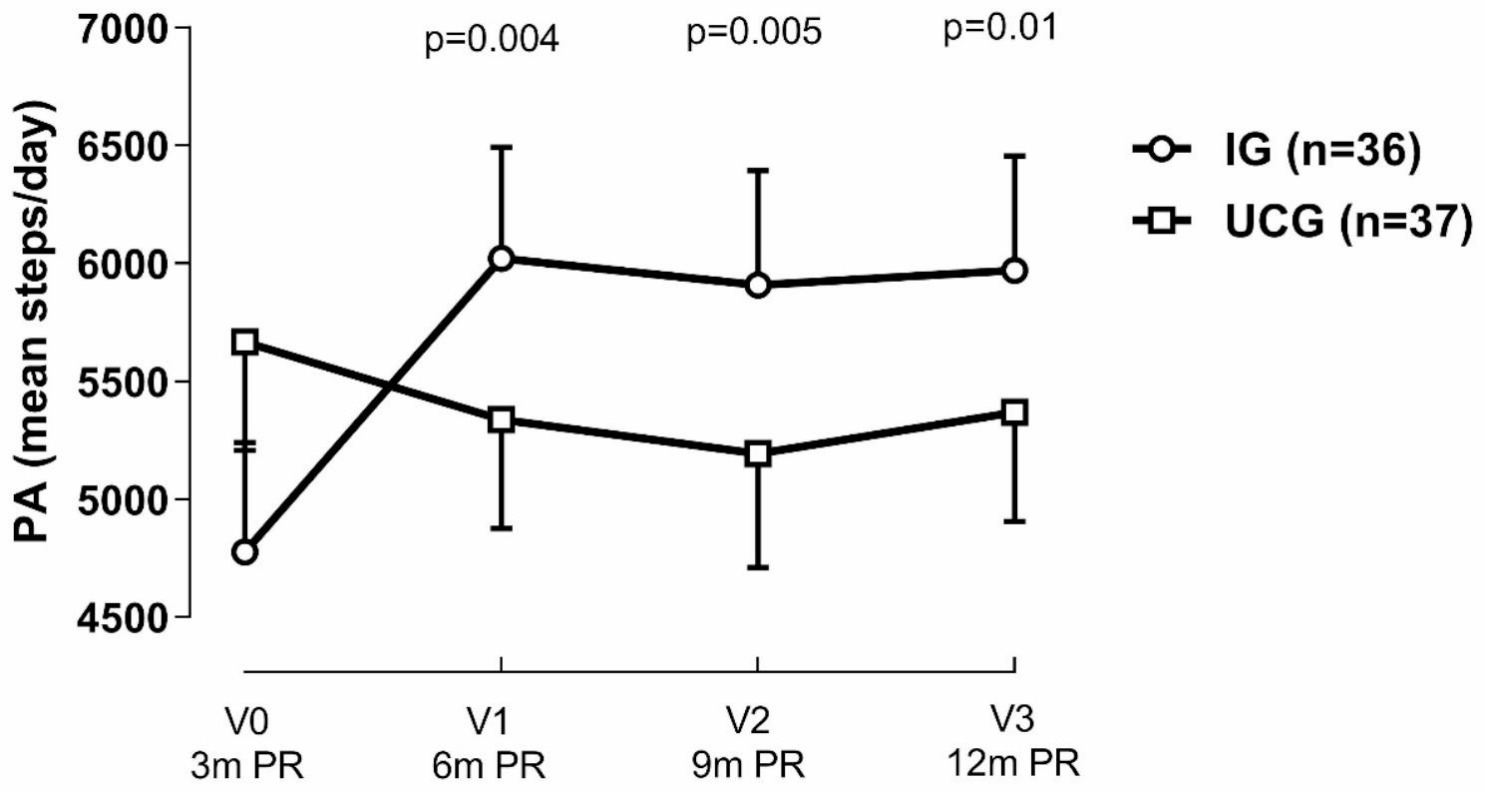
Comparison of the changes in PA between patients from both the IG and UCG, adjusted for daylight at each visit point. p-values indicate the interaction effect (with V0 as the reference). PA; physical activity; IG, intervention group; UCG, usual care group; n, number; m, months; PR, pulmonary rehabilitation. Data based on multiple imputation

Patients who received the PA-telecoaching intervention presented clinically important improvements in steps/day compared to patients in the UCG, at each time point after randomization (Fig. 3). Changes in time spent in MVPA and movement intensity during walking were not significantly different between groups at the end of follow-up (Table 2). The sensitivity analyses based on the complete dataset provided very similar results (Additional file 4).

**Table 2:** Comparison of changes in PA between IG and UCG groups. V0 was considered the reference at mixed model analysis

	V0	V1	V2	V3
**PA (steps/day)**				
UCG	Ref	-313 ± 315	-462 ± 342	-278 ± 318
IG		1277 ± 433	1228 ± 453	1177 ± 457
Between groups changes		1544 ± 534	1605 ± 570	1431 ± 555
p value		0.004	0.005	0.01
**Time spent in MVPA (min/day)**				
UCG	Ref	-3 ± 5	-4 ± 5	-0 ± 5
IG		11 ± 6	13 ± 6	10 ± 6
Between groups changes		13 ± 8	16 ± 8	10 ± 8
p value		0.0802	0.0466	0.2107
**Movement intensity (m/s** ^**2**^ **)**				
UCG	Ref	-0.018 ± 0.024	0.011 ± 0.027	0.001 ± 0.024
IG		0.057 ± 0.037	0.058 ± 0.040	-0.001 ± 0.039
Between groups changes		0.07 ± 0.04	0.04 ± 0.05	-0.01 ± 0.04
p value		0.0869	0.3599	0.8840

Values presented as estimates ± standard error obtained from the mixed model analysis adjusted for daylight. PA, physical activity; MVPA, moderate to vigorous PA. UCG, usual care group; IG, intervention group; min; minutes; m, meter; s, seconds. In case of missing data (for steps/day and MVPA) from the Dynaport Activity Monitor, multiple imputation was performed with the data from the Actigraph as outlined in the methodology section. At V1, V2 and V3, interaction effect with V0 as reference value are displayed

### Effect on secondary outcomes

The PA-telecoaching program did not induce significant improvements in terms of maximal, endurance and functional exercise tolerance, muscle strength and QoL as compared to usual care (Table 3 & Additional file 5), at none of the timepoints.

**Table 3:** Comparison of the changes in secondary outcomes between IG and UCG

	V0	V1	V2	V3
**VO**_**2**_ **peak (ml/min/kg)**				
UCG	Ref	-0.3 ± 0.5		-0.6 ± 0.5
IG		-1.0 ± 0.5		-1.3 ± 0.5
Between groups changes		-0.70 ± 0.75		-0.66 ± 0.76
p value		0.3525		0.3890
**Endurance duration CWRT (s)**				
UCG	Ref	4 ± 33		-64 ± 33
IG		43 ± 41		21 ± 42
Between groups changes		35 ± 52		87 ± 53
p value		0.5033		0.1039
**6MWD (meters)**				
UCG	Ref	-7 ± 8	-34 ± 8	-26 ± 8
IG		-6 ± 8	-28 ± 8	-20 ± 8
Between groups changes		2 ± 11	6 ± 12	7 ± 11
p value		0.8903	0.5923	0.5327
**Max isom QF (Nm/kg)**				
UCG	Ref	-0.01 ± 0.06	0.04 ± 0.07	0.02 ± 0.06
IG		-0.04 ± 0.05	0.01 ± 0.05	-0.06 ± 0.05
Between groups changes		-0.05 ± 0.08	-0.04 ± 0.08	-0.08 ± 0.80
p value		0.5543	0.6445	0.3037
**CRDQ**_**dyspnea (points)**_				
UCG	Ref	2 ± 1		0.5 ± 1
IG		4 ± 1		1 ± 1
Between groups changes		1.58 ± 1.15		0.58 ± 1.18
p value		0.1722		0.6232
**CRDQ**_**total (points)**_				
UCG	Ref	3.3 ± 2		-2 ± 2
IG		7.4 ± 2		-0.4 ± 2
Between groups changes		4.05 ± 2.95		1.81 ± 3.09
p value		0.1727		0.5603

Values presented as estimates ± standard error. m- months, PR, pulmonary rehabilitation program; VO_2_, oxygen uptake; CWRT, constant work rate test; 6MWD, six minutes walking distance; isom, isometric; QF, quadriceps force; CRDQ, Chronic Respiratory Disease Questionnaire; VO_2_peak, peak oxygen uptake; ml, millilitres; min, minutes; kg, kilogram; s, seconds Isom, isometric; Nm, Newton meters. At V1, V2 and V3, interaction effect with V0 as reference value are displayed in this table

### Effect on exploratory outcomes

The effect of the PA-telecoaching program on exploratory outcomes is displayed in Table 4. No significant differences between patients in the IG and the UCG group in terms of body weight, Bone mineral density (BMD) or markers of osteoporosis were observed. Similarly, no difference was found in blood markers except for a trend towards a reduction in fasted blood glucose favoring the IG group (p = 0.06; Table 4). A similar number of patients of both the IG and UCG group experienced MID in functional exercise tolerance, symptoms of dyspnea and QoL [23] from V-1 to V3 (Additional file 6).

**Table 4:** Effect of PA-telecoaching intervention on exploratory outcomes

	UCG Mean (SD)	IG Mean (SD)	Between group Mean(SE)	P value
**Bone Mineral Density (DEXA scan)**				
Δ T-Score total body	0 ± 0.37	0 ± 0.29	-0.09 (0.11)	0.4231
Δ T-Score femoral neck	-0.02 ± 0.21	-0.11 ± 0.38	-0.06 (0.08)	0.4789
**Fasting Blood profile**				
Δ Glucose (mg/dL)	1 ± 11	-9 ± 18	-6.80 (3.5)	0.0569
Δ Insulin (mg/dL)	-12 ± 45	0 ± 46	15 (13)	0.2676
Δ Triglycerides (mg/dL)	-7 ± 33	-1 ± 40	7.9 (8.6)	0.3668
Δ HDL cholesterol (mg/dL)	0 ± 9	2 ± 12	1.10 (2.9)	0.7070
Δ LDL cholesterol (mg/dL)	8 ± 18	-2 ± 26	-8.5 (6.7)	0.2085

Data are expressed as mean ± standard deviation. UCG, usual care group; IG, intervention group; DEXA, dual energy X-ray absorptiometry; g, gram; mg, milligram; dL, deciliters; HDL, high-density lipoprotein; LDL, low-density lipoprotein. Between group differences and p-values are based on ANVOCA analyses and expressed as mean ± SE.

### Adverse events and follow-up

Twenty patients (57%) experienced at least one (moderate or severe) acute exacerbation during the study in the UCG compared to 14 (47%) patients in the IG (p = 0.46). A total of 51 non-respiratory adverse events were reported during the study period, of which 20 (39%) occurred in the IG and 31 (61%) in the UCG. 47% of them led to a hospital admission. The adverse events were cardiovascular (25%), musculoskeletal (24%), internal (4%), cancer (2%) and other (45%). Two (swollen knee in UCG, joint pain in IG) were considered as “unlikely” related to the study, the other as “not” related to the study. One event (viral gastroenteritis) led to study discontinuation.

After finishing 6 months of PR and 9 months after randomization, slightly more (65%) patients from the UCG continued supervised training (i.e. typically 1x/week in primary/community care) compared to the IG (42% p = 0.14).

## Discussion

The semi-automated PA-telecoaching program implemented at 3 months of PR was able to induce a sustained and clinically significant [29] improvement in the amount of PA (i.e. steps/day) in patients with COPD. Other clinical benefits in physical fitness, symptoms and QoL were not better maintained in the IG compared to the UCG, 9 months after initiation of the PA telecoaching intervention.

Despite the ATS/ERS recommendation of including physical activity promotion within PR [8], it is still not clear how this is best implemented [30]. Real-time feedback combined with individual target setting are considered important components in interventions to change general behaviour [31], including PA [32, 33]. Previous studies showed conflicting results regarding change in PA when adding PA coaching among patients with COPD undergoing PR [13–18]. In contrast to our findings, four of these studies demonstrated that PA coaching interventions including PA counselling and/or step counter-based targets embedded in a PR program did not improve PA in the short [13, 16, 34] or long-term [1, 34].

A first explanation for this apparent discrepancy across trials, is the timing of the PA-coaching intervention. Providing PA coaching interventions at the start of a PR program might be overwhelming and unrealistic in these symptomatic, inactive and deconditioned patients [13, 16, 34]. In patients with better preserved exercise tolerance (i.e. 6MWD of 494 m [15] vs. range of 395–448 m on the 6MWD in the other trials [13, 14, 16, 34], a PA coaching intervention including both PA-counseling and step-counter based targets was effective started together with PR [15]. The level of exercise tolerance is a second factor that might explain conflicting findings across studies. Indeed, a better preserved exercise tolerance has been previously shown to improve the likelihood of PA increase [19]. As patients who are referred to PR present limiting functional exercise tolerance [35], our study was designed to implement the PA-telecoaching intervention at 3 months of PR, allowing prior improvement for exercise tolerance before the start of the PA coaching. The magnitude of the intervention effect was in line with a previous large multicentre study in stable patients not enrolled in PR program [9] and a recently published RCT starting coaching after a short inpatient-PR [18].

The present study is supported by the “Capability, Opportunity & Motivation” for Behaviour change (COM-B) system, a framework for understanding behaviour (change) from Michie and colleagues(36). In terms of ‘capability’, our design (starting after an initial period of PR only) allowed optimization of physical fitness (capacity) during the initial three months of PR (i.e. before start of the PA-telecoaching). ‘Opportunity’ (to become physically active) was addressed at the interview in the IG before the start and was further supported by telephone contacts during the intervention during which barriers and facilitators were discussed. ’Motivation’ was targeted with automatic messages which patients received on their smartphone (daily display of activity goals, daily and weekly feedback, cumulative achievements and positive tips to keep active) and telephone contacts. Furthermore, being monitored and supported by the investigators was considered an important motivation tool, based on previous patients’ feedback on this PA-telecoaching intervention [21].

Despite the significant effect on the amount of PA, this intervention did not result in sustained training benefits. The latter could have been expected as several of these PR training benefits are known to be positively associated with PA in patients with COPD [37]. The end points of exercise tolerance, muscle force, dyspnea symptoms and Qol that were initially enhanced with PR were not different between groups at the follow-up visit and a comparable and significant proportion of patients from both groups presented minimal important improvements of these end points at follow-up. Thus, the clinically relevant increase in step count [29], in absence of significant improvements in PA intensity, did not prevent patients to lose PR-benefits over time. Some possible factors explaining these findings are: (1) the telecoaching intervention was not able to prevent exacerbations, known to impact on physical fitness, QoL and symptoms; (2) This PA-telecoaching intervention focused primarily on increasing the number of steps/day using the step counter to provide direct feedback. This measure is a relevant PA outcome which is easy to measure and well understood by patients [38, 39]. While the intervention improved the number of steps per day, there was little cross over to enhanced physical fitness. Using steps as motivation does not include incentives towards intensity of the PA. In line with the principles of exercise training, in order to achieve significant physiologic benefits, progressive overload (intensity) has been proven to be important [40]. While it is possible that the effect size (0.30 at V3 and 0.34 at V1 for step count) is too small to render significant physiologic effect, it is more plausible to speculate that, to maintain benefits of PR, focus on intensity needs to be implemented in future studies. The PA intervention by Mendoza et al. [32], although achieving larger effects on PA (effect size of 0.48), did not lead to clinically relevant improvements in functional exercise tolerance (6MWD), either. Furthermore, a recent study suggests that this can be achieved by embedding exercise training (e.g. in the form of a set of exercises embedded in the coaching app) would allow to achieve maintained fitness, on top of improved physical activity [18]; and (3) Numerically more patients from the UCG continued supervised exercise training in other facilities after PR-discharge. This could partially contribute to the lack of between-group differences because they could continue to perform systematically planned exercises (cfr. progressive overload). Our data corroborate with others [18] also reporting no additional benefits on exercise tolerance when adding telecoaching and home exercises after PR. Interestingly, the latter study did report a better health status at follow-up in patients following the telecoaching intervention. The addition of a self-directed strength training program on top of PA goal setting might partly explain this difference. Importantly, the analyses on the impact on PR-benefits is a secondary outcome of this study. The present study was powered to detect a change in PA. Further larger studies are needed to confirm our findings, but from our data there may be more merit in integrating PA at sufficient intensity rather than to introduce only larger volumes of PA if maintenance of physiologic benefits is the goal.

In line with the results on exercise tolerance, muscle force, quality of life, body composition, BMD and blood markers of insulin and lipids were not clinically better in the IG at the end of the trial. A decrease in the level of fasted blood glucose in the IG was noted compared to the UCG, which could lower the incidence risk for diabetes [41]. The latter decrease was numerically higher than the decrease in fasted blood glucose levels reported from diet and PA-promotion programs in adults at risk of type 2 diabetes (mean [95% Confidence Intervals]-2.2 mg/dl [− 3.6 to − 0.9 mg/dL] [41]. However, this finding needs replication in a larger study. Increasing PA levels, nevertheless, remains an important health advice in any patient at risk of developing type II diabetes and PA-telecoaching can be a useful tool in this context.

### Strengths and limitations

To the best of our knowledge, this trial is one of the first to test a long-term effect of a PA intervention and to investigate the causal relationship between PA and PR-benefits using an interventional design after 3 months of PR and 9 months of follow-up. It therefore helps to understand the importance of achieving the long-term goals of PR as described in the guidelines for PR by major respiratory Societies [8]. The present PA-telecoaching intervention was specifically tailored to patients with COPD and took several aspects of intervention implementation into account which were reported in the previous RCT [21].

Due to the nature of the intervention, patients could not be blinded for group allocation. PR-sessions were performed in group and contamination could not be prevented. This study was conducted in a 6-month outpatient PR setting. This needs to be considered for the external validity of our findings. However, the actual PA-telecoaching intervention started after 3 months of PR. The study by Spielmanns et al. is a first external validation that the concept of starting PA coaching after shorter PR may also be successful [18]. The results related to changes from 0 months to 12 months of PR in clinical outcomes often related to PA were exploratory and not corrected for multiple testing and, therefore, should be interpreted with caution and confirmed in further prospective studies.

## Conclusion

The implementation of a semi-automated PA-telecoaching program after a 3 months-PR program was effective to improve the amount of PA. Contrary to our hypothesis, this did not result in a better maintenance of other PR-acquired benefits. Further studies need to look into further strategies to maintain the overall physical fitness benefits of PR as steps-guided PA-telecoaching may not suffice to achieve this goal.

### Electronic supplementary material

Below is the link to the electronic supplementary material.

**Additional file 1**. Methods detailed information and Figure AF1 (Algorithm for the calculation of the PA weekly goal in steps/day).

**Additional file 2**. Additional patient characteristics at randomization.

**Additional file 3**. Patient characteristics at the start of pulmonary rehabilitation.

**Additional file 4**. Sensitivity analysis based on complete dataset - Comparison of the changes in PA between intervention and usual care groups.

**Additional file 5**. Changes in additional secondary outcomes between intervention and usual care groups.

**Additional file 6**. Proportion long-term success vs no long-term success in terms of A) functional exercise tolerance, B) symptoms of dyspnea and C) Quality of Life.

**Additional file 7**. Consort checklist.

**Additional file 8**. TIDieR checklist.

## Data Availability

The datasets generated and/or analysed during the current study are not publicly available but are available from the corresponding author on reasonable request.

## Abbreviations

ACT: Actigraph
ATS: American Thoracic Society
BMD: Bone Mineral Density
COPD: Chronic Obstructive Pulmonary Disease
CPET: Cardiopulmonary Exercise Testing
CRDQ: Chronic Respiratory Disease Questionnaire
DAM: Dynaport Movemonitor
DEXA: Dual Energy X-ray Absorptiometry
ERS: European Respiratory Society
HDL: High-Density Lipoprotein
IG: Intervention Group
LDL: Low-Density Lipoprotein
MI: Movement Intensity
MID: Minimal Important Difference
mMRC: modified Medical Research Council
MVPA: Moderate to Vigorous Physical Activity
PA: Physical Activity
PR: Pulmonary Rehabilitation
RCT: Randomized Controlled Trial
QF: Quadriceps Force
QoL: Quality of Life
SD: Standard Deviation
STEP: Sustaining Training Effects through Physical activity promotion
UCG: Usual Care Group
V: Visit
WR: Work Rate
6MWD: Six Minutes Walking Distance
