# Supplementary material for: Sustaining training effects through physical activity coaching (STEP): a randomized controlled trial
Source: Int J Behav Nutr Phys Act. 2023 Oct 10;20:121. doi: 10.1186/s12966-023-01519-w (PMC10563200; doi:10.1186/s12966-023-01519-w)
Supplement: Supplementary file 1 — Additional file 1. Methods detailed information and Figure AF1 (Algorithm for the calculation of the PA weekly goal in steps/day). [file 12966_2023_1519_MOESM1_ESM.docx]

**Additional file 1. Methods detailed information**

**Subjects**

The following inclusion and exclusion criteria were used for the trial:

*Inclusion criteria:*

- Having a primary diagnosis of COPD (post-bronchodilator: FEV_1_/FVC < 70%)
- Having at least 40 years of age
- Having smoked at least 10 pack years
- Having completed 3 months of pulmonary rehabilitation (PR) at the Leuven Hospital
- Having the cognitive ability to manage electronic devices and having access to internet signal at home

*Exclusion criteria:*

- Having predominant respiratory diseases other than COPD
- Having important comorbidities (i.e. orthopedic, neurological or muscular) that interfere with the normal biomechanical movement patters and which do not allow patients to increase their physical activity (PA)
- Not willing to continue for another 3 months of PR at the Leuven Hospital
- Clinical instability during inclusion interfering with PA measurement and continuation of the PR
- Having psychiatric issues interfering with the PR delivery at V0
- Participating in other interventional studies which could interfere with measurements throughout the trial
- Following PR prior to lung transplantation or lung volume reduction surgery

**Randomization details**

The randomization (1:1) was generated through opaque sealed envelopes, prepared in blocks of four and six by non-study staff and stratified by functional exercise tolerance at inclusion (<480 m vs. ≥480m) as patients with lower exercise tolerance are less likely to obtain clinically important PA improvements(1). The cut-off of 480 meters was based on a median split of all available six-minute walking distances (6MWD) at 3 months of PR in our PR database.

**Detailed description of the semi-automated PA telecoaching intervention**

The intervention implemented several principles of behavior change which are included in the taxonomy of Michie, et al. such as self-monitoring, goal setting, feedback and problem solving(2). Patients were instructed to wear the step counter during waking hours and to use the smartphone application on a daily basis. The smartphone application ensured transmission of step data onto the central server to allow semi-automatic coaching and verification by the investigators. In addition, it provided patients with daily goals, feedback and educational messages in attempt to enhance their motivation, commitment and self-efficacy. Patients received training in the usage of the smartphone and the step counter. At any time during the intervention period, patients were allowed to contact the study investigators for technical issues related to both interventional devices. Patients used the smartphone application from V0 until V2. From there on (i.e. final 3 months of the STEP trial – V2 to V3), patients were asked to continue using the step counter alone together with the final step count goal they had agreed with their coaches as their personal target.

The goal calculation algorithm used in the MrPAPP trial(3) was adjusted by considering findings of the patients’ interviews on the acceptability of the previously tested intervention(4). The algorithm for the calculation of the goal in the present trial is displayed in Figure AF1. During the first week, patients were not provided with a goal but were asked to wear the step counter and send their steps via Bluetooth to the smartphone application. After collecting at least 6 days of steps, patients received their first goal on the next Monday morning, which equaled the median of the six most active days. Afterwards, the goal calculations for all weeks throughout the trial followed the same procedure:

1. If patients reached their goal on at least 4 days during the week, they were considered to have reached their goal. If their goal was at least 1000 steps more than the goal of the previous week they were asked “Do you want to increase your goal next week” via 3 options on the smartphone display: a) ‘yes, by 10 minutes’ b) ‘yes, by 5 minutes’ c) ‘no, I do not want to increase’. If their goal was less than 1000 steps more than the goal of the previous week, patients received the same question, however, without option of increasing with 10 minutes
2. If patients reached their goal on 2 or 3 days during their week, the median value of steps/day was calculated for that week. If this value was higher than the target they were considered to have reached their goal (cfr. paragraph above). If the median was less than the target, they received a mark in the system and their goal was unchanged. If a patient receives a mark for two consecutive weeks, the goal was reduced (cfr. coming paragraph).
3. If patients reached their goal on 0 or 1 day that week, their goal was reduced. The value of the previous goal – 200 was compared to the median value of that week + 500 and the new goal was considered to be the lowest value of the latter two. However, if more than 60% of the patients being coached in the IG reached their goal not more than 1 day per week than the goal was automatically maintained for all these patients for that specific week to account for general difficulties caused by for example the weather condition.

The study investigators who were the coaches for this intervention (ML, FMR, AB) were physiotherapists with experience in pulmonary rehabilitation. They had been trained by the research group members who had previous experience with the delivery of this intervention (TT, HD).

**Detailed description of the outcome measures**

*Patient characterization*

Lung function, lung volumes and diffusion capacity measurements (V0) were performed according to European Respiratory Society recommendations(5) and compared to normal values reported by Quanjer et al(6).

At V0, all included patients were asked during their interview to rate on a 0 to 10 Likert scale how important their perception was to 1) increase or maintain an active lifestyle during and after the PR (i.e. motivation) and 2) how confident they felt in reaching the latter aim (i.e. self-efficacy). The higher the score, the better the patients’ motivation and self-efficacy.

*Secondary outcomes*

Maximal exercise tolerance (V0, V1, V3) was assessed by a maximal incremental cycling test(7), taking peak oxygen uptake corrected for weight (relative VO_2_peak) as the main outcome. Reference values were those reported by Jones et al(8).

Endurance tolerance (V0, V1, V3) was assessed by a constant work rate test (CWRT) until symptom limitation at a work rate of 75% of the maximal WR obtained at the maximal incremental cycling test at V0 (Ergometrics 900, Ergoline, Bitz, Germany)(9). If the CWRT lasted more than 480 or less than 180 seconds, a new CWRT was performed, within the same week with an adjusted work rate (9). Endurance duration was the main outcome and minute ventilation, heart rate and reported fatigue and dyspnea (Borg scale) at isotime were also retrieved.

Functional exercise tolerance (V0, V1, V2, V3) was assessed through a six minutes walking test (6MWT), according to the American Thoracic Society/European Respiratory Society guidelines at each visit of the trial with the distance achieved being the main outcome (six-minute walking distance (6MWD))(10). Saturation and heart rate were continuously monitored, while Borg scores for dyspnea and fatigue were assessed at the start and right after the end of the 6MWT. The best of two tests was used for analysis and compared with reference values(11). The use of oxygen during the 6MWT, when necessary, was standardized throughout the trial.

Quadriceps force (QF) measurements (V0, V1, V2, V3) were obtained by isometric maximal voluntary contraction against a fixed strain gauge (SG) with signal analogue force transducer (546QD; CDS Milan, Italy) and amplifier (Biopac MP150; Biopac Systems, Goleta, California, USA) (12).

The interview-based version of the Chronic Respiratory Disease Questionnaire (CRDQ) (13) was completed at V0, V1 and V3. Symptoms of dyspnea were obtained from the specific domain of this questionnaire, with scores ranging from 0 to 35. Quality of life was the total score, ranging from 0 to 140. Better scores of this instrument indicate better outcomes.

At V-1 (start of PR) and V3, a dual energy X-ray absorptiometry (DEXA) scan (QDR 4500A, Discovey scanners, Hologic, Inc., Bedford, MA, USA) was performed, which provided information bone mineral density (total body T-score and femoral neck T-score). A fasted blood sample was collected at V-1 and V3 and insulin, glucose, HDL and LDL were analysed.

Information regarding acute exacerbations (AE) was collected at each visit based on patients’ self- report. AE was defined as an increase in respiratory symptoms that caused the need for treatment with antibiotics or oral corticosteroids (i.e. mild AE) and in case patients were hospitalized due to respiratory-related reasons (i.e. severe AE)(14).

Figure AF1.


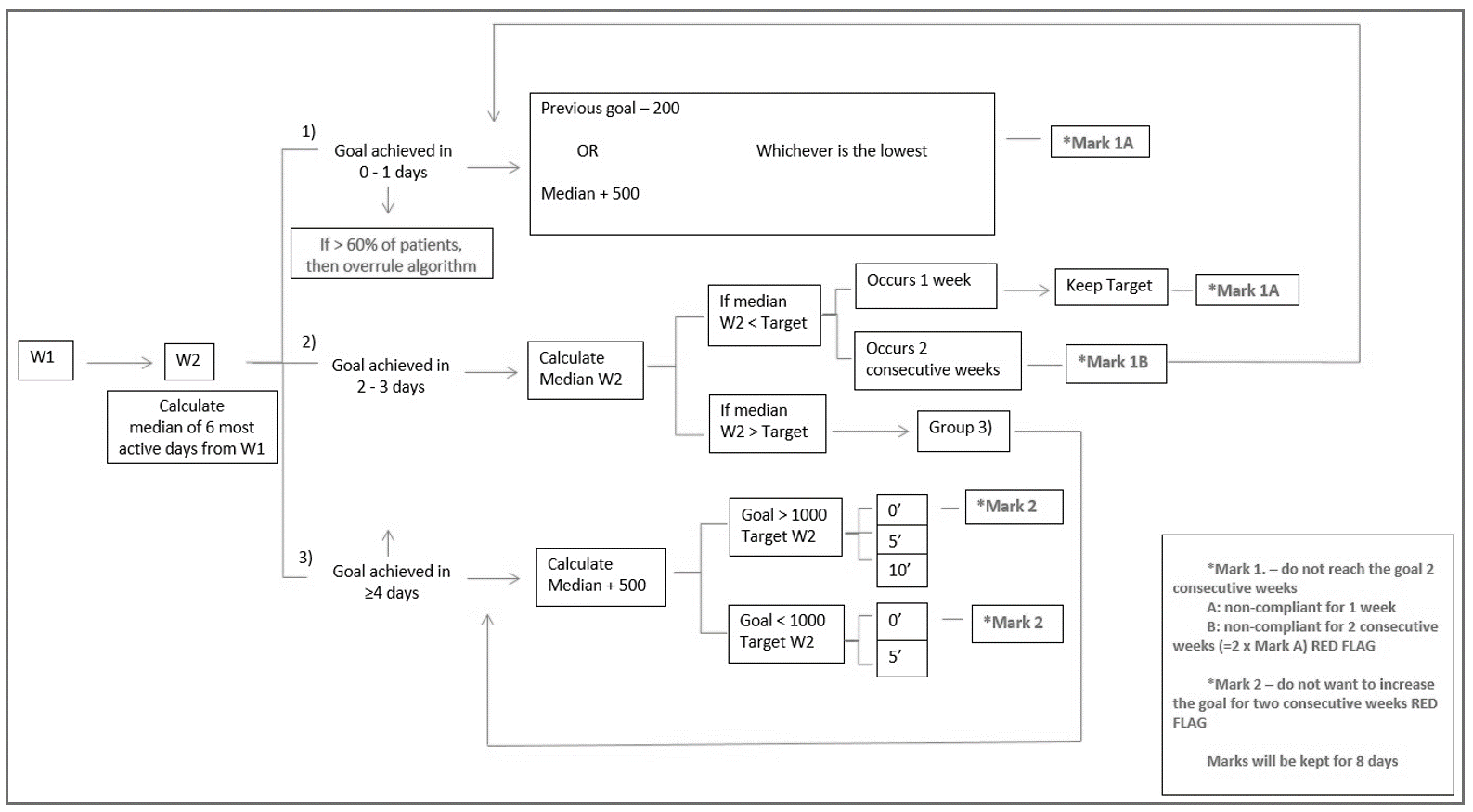


Figure AF1. Algorithm for the calculation of the PA weekly goal in steps/day. W, Week; ‘ minutes

**REFERENCE LIST**

1. Osadnik CR, Loeckx M, Louvaris Z, Demeyer H, Langer D, Rodrigues FM, et al. The likelihood of improving physical activity after pulmonary rehabilitation is increased in patients with COPD who have better exercise tolerance. Int J COPD. 2018;13:3515-3527.

2. Michie S, Richardson M, Johnston M, Abraham C, Francis J, Hardeman W, et al. The behavior change technique taxonomy (v1) of 93 hierarchically clustered techniques: Building an international consensus for the reporting of behavior change interventions. Ann Behav Med. 2013;46:81–95.

3. Demeyer H, Louvaris Z, Frei A, Rabinovich RA, De Jong C, Gimeno-Santos E, et al. Physical activity is increased by a 12-week semiautomated telecoaching programme in patients with COPD: A multicentre randomised controlled trial. Thorax. 2017;72:415–23.

4. Loeckx M, Rabinovich RA, Demeyer H, Louvaris Z, Tanner R, Rubio N, et al. Smartphone-Based Physical Activity Telecoaching in Chronic Obstructive Pulmonary Disease: Mixed-Methods Study on Patient Experiences and Lessons for Implementation. JMIR Mhealth Uhealth 2018;6:e200.

5. Graham BL, Brusasco V, Burgos F, Cooper BG, Jensen R, Kendrick A, et al. Executive Summary: 2017 ERS/ATS standards for single-breath carbon monoxide uptake in the lung. Eur Respir J. 2017;49:16E0016.

6. Quanjer P., Tammeling GJ, Cotes JE, Pedersen OF, Peslin R, Yernault JC. Lung volumes and forced ventilatory flows. Eur Respir J. 1993;6:5– 40.

7. Rodrigues FM, Loeckx M, Hornikx M, Van Remoortel H, Louvaris Z, Demeyer H, et al. Six years progression of exercise capacity in subjects with mild to moderate airflow obstruction, smoking and never smoking controls. PLoS One. 2018;13:e0208841.

8. Jones NL, Makrides L, Hitchcock C, Chypchar T NM. Normal standards for an incremental progressive cycle ergometer test. Am Rev Respir Dis. 1985;May;13:700–8.

9. Puente-Maestu L, Palange P, Casaburi R, Laveneziana P, Maltais F, Neder JA, et al. Use of exercise testing in the evaluation of interventional efficacy: an official ERS statement. Eur Respir J. 2016;47:429 LP – 460.

10. Holland AE, Spruit MA, Troosters T, Puhan MA, Pepin V, Saey D, et al. An official European Respiratory Society/American Thoracic Society technical standard: field walking tests in chronic respiratory disease. Eur Respir J. 2014;44:1428– 1446.

11. Troosters T, Gosselink R, Decramer M. Six minute walking distance in healthy elderly subjects. Eur Respir J. 1999;14:270 LP – 274.

12. Machado Rodrigues F, Demeyer H, Hornikx M, Camillo CA, Calik-Kutukcu E, Burtin C, et al. Validity and reliability of strain gauge measurement of volitional quadriceps force in patients with COPD. Chron Respir Dis 2017;14:289-297.

13. Guyatt GH, Berman LB, Townsend M, Pugsley SO, Chambers LW. A measure of quality of life for clinical trials in chronic lung disease. Thorax. 1987;42:773 LP – 778.

14. Wedzicha JA, Seemungal TAR. COPD exacerbations: defining their cause and prevention. Lancet (London, England). 2007;370:786–96.
