# Supplementary material for: Sustaining training effects through physical activity coaching (STEP): a randomized controlled trial
Source: Int J Behav Nutr Phys Act. 2023 Oct 10;20:121. doi: 10.1186/s12966-023-01519-w (PMC10563200; doi:10.1186/s12966-023-01519-w)
Supplement: Supplementary file 2 — Additional file 2. Additional patient characteristics at randomization. [file 12966_2023_1519_MOESM2_ESM.docx]

**Additional file 2. Additional patient characteristics at randomization**

**Table AF2.** Additional patient characteristics at randomization

|  | **UCG (n=37)** | **IG (n=36)** |
| --- | --- | --- |
| **FEV_1_ (l)** | 1.34 ± 0.61 | 1.33 ± 0.51 |
| **RV/TLC** | 0.52 ± 0.11 | 0.53 ± 0.11 |
| **FRC (l)** | 4.88 ± 1.22 | 5.25 ± 1.62 |
| **Peak WR maximal CPET (W)** | 73 ± 27 | 78 ± 30 |
| **WR on CWRT (W)** | 58 ± 22 | 60 ± 25 |
| **CRDQ_fatigue_** | 19 ± 4 | 19 ± 3 |
| **CRDQ_emotion_** | 32 ± 5 | 32 ± 6 |
| **CRDQ_mastery_** | 20 ± 4 | 18 ± 4 |
| **Living alone (Yes), n (%)** | 14% | 17% |
| **Active working (Yes), n (%)** | 14% | 25% |
| **Education, n (%)**  **None**  **Primary school**  **Secondary school**  **Further education** | 3%  6%  57%  34% | 0%  13%  47%  40% |

Values presented as mean ± standard deviation or as number (percentage). UCG, usual care group; IG, intervention group; n, number of patients; FEV_1_, forced expiratory volume in 1 second; l, liter, TL_CO_, diffusing capacity for carbon monoxide; RV, residual volume; TLC, total lung capacity; FRC, functional residual capacity; WR, work rate; CPET, cardiopulmonary exercise testing; W, Watts; CWRT, constant work rate test; CRDQ, Chronic Respiratory Disease Questionnaire.
