# Supplementary material for: Sustaining training effects through physical activity coaching (STEP): a randomized controlled trial
Source: Int J Behav Nutr Phys Act. 2023 Oct 10;20:121. doi: 10.1186/s12966-023-01519-w (PMC10563200; doi:10.1186/s12966-023-01519-w)
Supplement: Supplementary file 3 — Additional file 3. Patient characteristics at the start of pulmonary rehabilitation. [file 12966_2023_1519_MOESM3_ESM.docx]

**Additional file 3. Patient characteristics at the start of pulmonary rehabilitation.**

**Table AF3.** Patient characteristics at the start of pulmonary rehabilitation.

|  | **UCG (n=37)** | **IG (n=36)** |
| --- | --- | --- |
| **BMI (kg/m^2^)** | 26 ± 6 | 25± 6 |
| **FEV_1_ (% pred)** | 49 ± 16 | 50 ± 22 |
| **Peak WR maximal CPET (W)** | 65 ± 25 | 66 ± 25 |
| **VO_2_peak (ml/min/kg)** | 14.3 ± 3.3 | 15.4 ± 2.9 |
| **VO_2_peak (% pred)** | 70 ± 20 | 68 ± 25 |
| **6MWD (meters)** | 452 ± 68 | 478 ± 81 |
| **6MWD (% pred)** | 73 ± 13 | 74 ± 10 |
| **Max isom QF (Nm)** | 121± 45 | 125 ± 47 |
| **Max isometric QF (Nm/kg)** | 1.69 ± 0.40 | 1.78 ± 0.51 |
| **CRDQ_dyspnea_** | 15 ± 4 | 16 ± 5 |
| **CRDQ_fatigue_** | 16 ± 4 | 16 ±4 |
| **CRDQ_emotion_** | 27 ± 5 | 28 ± 6 |
| **CRDQ_mastery_** | 17 ± 5 | 17 ± 5 |
| **CRDQ_total_** | 75 ± 11 | 76 ± 13 |
| **PA (steps/day)** | 4678 ± 2344 | 4164 ± 1947 |
| **T-Score total body** | -2.06 ± 1.16 | -1.29 ± 1.44 |
| **T-Score femoral neck** | -2.22 ± 0.84 | -1.92 ±0.93 |
| **Glucose (mg/dl)** | 102 ± 11 | 106 ± 20 |
| **Insulin (mg/dl)** | 88 ± 64 | 97 ± 65 |
| **Triglycerides (mg/dl)** | 104 ± 39 | 121 ± 55 |
| **HDL cholesterol (mg/dl)** | 67 ± 23 | 60 ± 16 |
| **LDL cholesterol (mg/dl)** | 91 ± 31 | 104 ± 38 |

Values presented as mean ± standard deviation or as number (percentage). UCG, usual care group; IG, intervention group; BMI, body mass index; kg, kilograms; FEV_1_, forced expiratory volume in 1 second; pred, predicted; WR, work rate; CPET, cardiopulmonary exercise testing; W, Watts; VO_2_, oxygen uptake; ml, millilitres; min, minutes; s, seconds; 6MWD, six minutes walking distance; Max, maximal; isom, isometric; QF, quadriceps force; Nm, Newton*meters; CRDQ, Chronic Respiratory Disease Questionnaire; PA, physical activity; g, gram; HDL, high density lipoproteins; LDL, low density lipoproteins.
