# Supplementary material for: Sustaining training effects through physical activity coaching (STEP): a randomized controlled trial
Source: Int J Behav Nutr Phys Act. 2023 Oct 10;20:121. doi: 10.1186/s12966-023-01519-w (PMC10563200; doi:10.1186/s12966-023-01519-w)
Supplement: Supplementary file 4 — Additional file 4. Sensitivity analysis based on complete dataset - Comparison of the changes in PA between intervention and usual care groups. [file 12966_2023_1519_MOESM4_ESM.docx]

**Additional file 4. Sensitivity analysis based on complete dataset – Comparison of the changes in PA between intervention and usual care groups.**

**Table AF4**. Sensitivity analysis based on complete dataset – Comparison of the changes in PA between IG and UCG groups.

|  | V0 | V1 | V2 | V3 |
| --- | --- | --- | --- | --- |
| **PA (steps/day)** |  |  |  |  |
| UCG | 5172 ± 346 | -315 ± 355 | -344 ± 398 | -233 ± 363 |
| IG |  | 1254 ± 479 | 1336 ± 518 | 965 ± 515 |
| Between groups changes | - | 1566 ± 590 | 1636 ± 650 | 1196 ± 619 |
| p value | - | 0.008 | 0.01 | 0.055 |
| **Time spent in MVPA (min)** |  |  |  |  |
| UCG | 83 ± 5 | -5 ± 4 | -6 ± 5 | -2 ± 6 |
| IG |  | 12 ± 6 | 15 ± 7 | 11 ± 7 |
| Between groups changes | - | 17 ± 8 | 20 ± 9 | 13 ± 8 |
| p value | - | 0.03 | 0.02 | 0.13 |

V0 was considered the reference in the mixed model analysis. Common baseline corrected for daylight indicated under V0 column.
