# Supplementary material for: Sustaining training effects through physical activity coaching (STEP): a randomized controlled trial
Source: Int J Behav Nutr Phys Act. 2023 Oct 10;20:121. doi: 10.1186/s12966-023-01519-w (PMC10563200; doi:10.1186/s12966-023-01519-w)
Supplement: Supplementary file 5 — Additional file 5. Changes in additional secondary outcomes between intervention and usual care groups. [file 12966_2023_1519_MOESM5_ESM.docx]

**Additional file 5. Changes in additional secondary outcomes between intervention and usual care groups**

**Table AF5.** Changes in additional secondary outcomes between intervention and usual care groups.

|  | V0 | V1 | V2 | V3 |
| --- | --- | --- | --- | --- |
| **Peak WR maximal CPET (W)** |  |  |  |  |
| Usual care group | 75 ± 3 | 0 ± 2 | - | -5 ± 2 |
| Intervention group |  | -4 ± 2 | - | -8 ± 2 |
| Between groups changes | - | -3.96 ± 3.12 | - | -3.75 ± 3.20 |
| p value |  | 0.2069 |  | 0.2426 |
| **VE at isotime CWRT (l/min)** |  |  |  |  |
| Usual care group | 40.2 ± 2.1 | 0.07 ± 0.8 | - | 1.3 ± 0.8 |
| Intervention group |  | -1.3 ± 0.9 | - | -2.5 ± 0.9 |
| Between groups changes | - | -1.5 ± 1.4 | - | -3.8 ± 1.4 |
| p value |  | 0.2710 |  | 0.0068 |
| **BORG - F at isotime CWRT** |  |  |  |  |
| Usual care group | 4.0 ± 0.3 | 0.8 ± 0.3 | - | 1.0 ± 0.3 |
| Intervention group |  | -0.7 ± 0.3 | - | 0.3 ± 0.3 |
| Between groups changes | - | -1.7 ± 0.6 | - | -0.6 ± 0.6 |
| p value |  | 0.0029 |  | 0.2664 |
| **BORG - D at isotime CWRT** |  |  |  |  |
| Usual care group | 4.2 ± 0.3 | 0.3 ± 0.4 | - | 0.9 ± 0.4 |
| Intervention group |  | -0.1 ± 0.4 | - | 0.7 ± 0.4 |
| Between groups changes,  p value | - | -0.8 ± 0.7 0.2506 | - | -0.3 ± 0.7 0.6427 |
| **Heart rate at isotime CWRT** |  |  |  |  |
| Usual care group | 96 ± 4 | 6 ± 2 | - | 7 ± 2 |
| Intervention group |  | 1 ± 3 | - | -6 ± 3 |
| Between groups changes | - | -6 ± 4 | - | -12 ± 4 |
| p value |  | 0.1640 |  | 0.0093 |
| **Max isom QF (Nm)** |  |  |  |  |
| Usual care group | 132 ± 5 | -1 ± 4 | 5 ± 4 | -2 ± 4 |
| Intervention group |  | -1 ± 4 | 1 ± 4 | -2 ± 4 |
| Between groups changes | - | -0.84 ± 5.36 | -1.85 ± 5.64 | -6.29 ± 5.46 |
| p value |  | 0.8758 | 0.7434 | 0.2507 |
| **CRDQ_fatigue_** |  |  |  |  |
| Usual care group | 19 ± 0.4 | 0.5 ±0.5 | - | -1.6 ± 0.5 |
| Intervention group |  | 0.6 ± 0.4 | - | -1.1 ± 0.5 |
| Between groups changes | - | 0.24 ± 0.83 | - | 0.40 ± 0.86 |
| P value |  | 0.7693 |  | 0.6416 |
| **CRDQ_emotion_** |  |  |  |  |
| Usual care group | 32 ± 0.7 | 1.0 ± 0.8 | - | -0.3 ± 0.8 |
| Intervention group |  | 1.8 ± 0.7 | - | -0.3 ± 0.8 |
| Between groups changes | - | 0.86 ± 1.31 | - | 0.04 ± 1.36 |
| p value |  | 0.5126 |  | 0.9743 |
| **CRDQ_mastery_** |  |  |  |  |
| Usual care group | 19 ± 0.5 | 0.8 ± 0.5 | - | -0.1 ± 0.5 |
| Intervention group |  | 1.3 ± 0.4 | - | 0.4 ±0.5 |
| Between groups changes | - | 0.61 ± 0.83 | - | 0.55 ± 0.85 |
| p value |  | 0.4652 |  | 0.5219 |

Values presented as estimates ± standard error.; WR, work rate; CPET, cardiopulmonary exercise testing; W, Watts; Ve, minute ventilation; CWRT, constant work rate test; BORG - F, fatigue score; BORG – D, dyspnea score; Max, maximal; isom, isometric; QF, quadriceps force; Nm; Newton*meter; CRDQ, Chronic Respiratory Disease Questionnaire. The values at V0 are the common baseline values of the variables in both groups. At V1, V2 and V3, relative values with V0 as reference value are displayed in this table.
