# Supplementary material for: Sustaining training effects through physical activity coaching (STEP): a randomized controlled trial
Source: Int J Behav Nutr Phys Act. 2023 Oct 10;20:121. doi: 10.1186/s12966-023-01519-w (PMC10563200; doi:10.1186/s12966-023-01519-w)
Supplement: Supplementary file 6 — Additional file 6. Proportion long-term success vs no long-term success in terms of A) functional exercise tolerance, B) symptoms of dyspnea and C) Quality of Life. [file 12966_2023_1519_MOESM6_ESM.docx]

**Additional file 6. Proportion of long-term success vs no long-term success in terms of A) functional exercise tolerance, B) symptoms of dyspnea and C) QoL.**

**Table AF6.** Proportion of long-term success vs no long-term success in terms of A) functional exercise tolerance, B) symptoms of dyspnea and C) QoL.

|  | MID | p-value |
| --- | --- | --- |
| **A) Functional exercise tolerance (6MWD)** |  |  |
| Number of patients (n, %) |  |  |
| UCG | 16 (46%) | 0.5655 |
| IG | 12 (39%) |  |
| **B) Symptoms of dyspnea (CRDQ_dyspnea_)** |  |  |
| Number of patients (n, %) |  |  |
| UCG | 25 (76%) | 0.4760 |
| IG | 21 (68%) |  |
| **C) QoL (CRDQ_total_)** |  |  |
| Number of patients (n, %) |  |  |
| UCG | 21 (72%) | 0.3489 |
| IG | 17 (61%) |  |

Values are presented as numbers (proportions). MID; minimal important difference; 6MWD, six minutes walking distance; QoL, quality of life; CRDQ, Chronic Respiratory Disease Questionnaire. MID for 6MWD = 30 meters, for CRDQ_dyspnea_ 2.5 points, for CRDQ_total_ 10 points(1). Patients who had missing values at V3 were excluded from this analysis.

Reference

1. Puhan MA, Lareau SC. Evidence-based outcomes from pulmonary rehabilitation in the chronic obstructive pulmonary disease patient. Clin Chest Med. 2014;35:295–301
